# Lower beta cell yield from donor pancreases after controlled circulatory death prevented by shortening acirculatory warm ischemia time and by using IGL-1 cold preservation solution

**DOI:** 10.1371/journal.pone.0251055

**Published:** 2021-05-03

**Authors:** Diedert L. De Paep, Freya Van Hulle, Zhidong Ling, Marian Vanhoeij, Jacques Pirenne, Bart Keymeulen, Daniel Pipeleers, Daniel Jacobs-Tulleneers-Thevissen

**Affiliations:** 1 Diabetes Research Center, Vrije Universiteit Brussel (VUB), Brussels, Belgium; 2 Diabetes Clinic, Vrije Universiteit Brussel (VUB), Universitair Ziekenhuis Brussel (UZ Brussel), Brussels, Belgium; 3 Department of Surgery, Vrije Universiteit Brussel (VUB), Universitair Ziekenhuis Brussel (UZ Brussel), Brussels, Belgium; 4 Department of Abdominal Transplantation and Transplantation Coordination, University Hospitals Leuven, Leuven, Belgium; Imperial College Healthcare NHS Trust, UNITED KINGDOM

## Abstract

Organs from donors after controlled circulatory death (DCD III) exhibit a higher risk for graft dysfunction due to an initial period of warm ischemia. This procurement condition can also affect the yield of beta cells in islet isolates from donor pancreases, and hence their use for transplantation. The present study uses data collected and generated by our Beta Cell Bank to compare the number of beta cells in isolates from DCD III (n = 141) with that from donors after brain death (DBD, n = 609), before and after culture, and examines the influence of donor and procurement variables. Beta cell number per DCD III-organ was significantly lower (58 x 10^6^ versus 84 x 10^6^ beta cells per DBD-organ; p < 0.001) but their purity (24% insulin positive cells) and insulin content (17 μg / 10^6^ beta cells in DCD III-organs versus 19 μg / 10^6^ beta cells in DBD-organs) were similar. Beta cell number correlated negatively with duration of acirculatory warm ischemia time above 10 min; for shorter acirculatory warm ischemia time, DCD III-organs did not exhibit a lower beta cell yield (74 x 10^6^ beta cells). Use of Institut Georges Lopez-1 cold preservation solution instead of University of Wisconsin solution or histidine-tryptophan-ketoglutarate also protected against the loss in beta cell yield from DCD III-organs (86 x 10^6^ for IGL-1 versus 54 x 10^6^ and 65 x 10^6^ beta cells respectively, p = 0.042). Multivariate analysis indicates that both limitation of acirculatory warm ischemia time and use of IGL-1 prevent the reduced beta cell yield in islet cell isolates from DCD III-organs.

## Introduction

Intraportal islet cell grafts prepared from human donor pancreases can correct hyperglycemia in type 1 diabetic patients provided that a sufficient functional beta cell mass is implanted [1–5]. Clinical islet transplant programs are however limited by scarcity of donor pancreases that yield a sufficiently large number of islets in their isolates. Despite prior donor selection, only half of the isolation procedures lead to a preparation that meets criteria for clinical transplantation [6]; moreover, most patients require multiple infusions [1, 3]. In our clinical program, deceased donor pancreases, including suboptimal organs are processed, and isolated fractions are cultured to remove damaged cells and contaminating exocrine cells. This produces preparations in which the number of beta cells is determined and used for combining multiple isolates to reach quantitative criteria [1, 7, 8]. It has already been shown that donors after circulatory death (DCD) can be used for islet isolation and transplantation when cessation of circulation occurs under controlled circumstances (Maastricht category III) [9], and could thus be considered as a supplementary supply of organs for islet cell transplantation [10, 11]. In this context, we have extended our organ donor pool with donors after controlled circulatory death. It is however not excluded that this procurement condition affects the number of beta cells. In general, DCD III organs exhibit a higher risk for posttransplant dysfunction due to an initial period of warm ischemia before starting cold preservation [12–14]. This risk discourages islet transplant programs to include DCD III organs for islet isolation or to use more stringent selection criteria then for donors after brain death (DBD) [15], despite previously reported comparisons between pancreases procured from DCD III and DBD that were indicative for a similar yield in isolated islets [10, 11, 16, 17]. While these DCD III cohorts were small and highly selected, the present study is conducted on a larger, more heterogenous series and directly determines the target criterium, the number of beta cells, and its correlation with donor and procurement variables.

## Materials and methods

### Study design

The database of our Beta Cell Bank was used to conduct a retrospective analysis of donor and procurement characteristics and associated quality control data of pancreatic isolates between 18/05/2007 and 20/12/2018. The study was reviewed and approved by the ethical committee of Universitair Ziekenhuis Brussel (BUN 143202042685); Consent for organ/tissue donation and clinical transplantation was obtained from all organ donors according to legislation in the country of organ procurement. Our ethical committee allowed use of anonymized data from the donor center for retrospective analysis.

### Organ allocation and procurement

**Fig 1:**
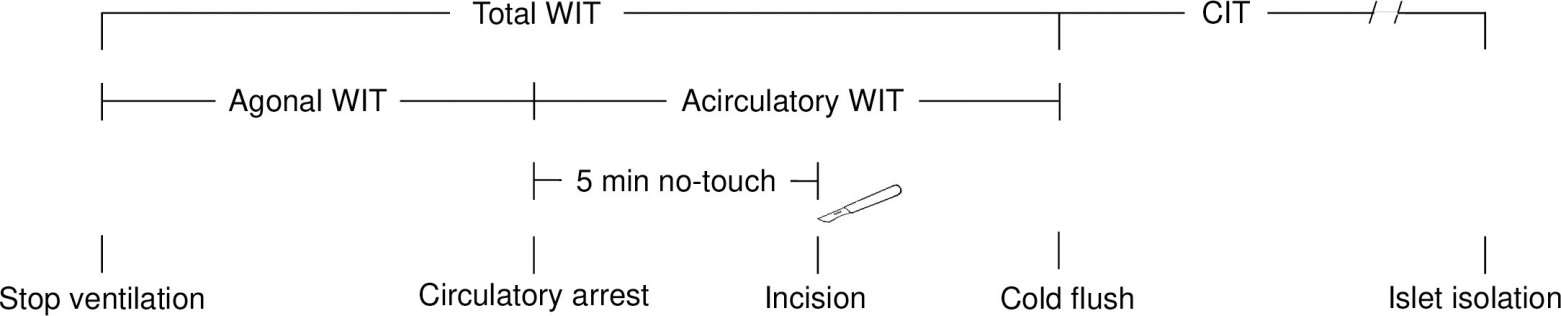
Definition of warm and cold ischemia times in donation after controlled circulatory death. Total warm ischemia time (WIT) represents the time between withdrawal of mechanical ventilation and abdominal aorta cannulation with initiation of cold preservation. It consists of an agonal phase (time between withdrawal of mechanical ventilation and circulatory arrest) and an acirculatory phase (time between circulatory arrest and abdominal aorta cannulation with initiation of cold flush). Cold ischemia time (CIT) starts with aortic flush using cold preservation solution and ends at start of islet isolation procedure. Pancreatectomy time is the time between cannulation and the moment the pancreas is preserved on ice outside the body.

Our facility for islet isolation receives pancreases from the Eurotransplant network (Leiden, the Netherlands). Pancreases are procured according to local practices by procurement teams of the transplant center affiliated to the donor hospital, and shipped to our facility using non-priority transportation. When applying the set inclusion criteria we identified 609 DBD and 141 DCD III (according to the Maastricht classification) [9] in our database. Donor, procurement characteristics and North American Islet Donor Scores [18] are listed in Table 1. Donor management and organ procurement was performed according to local practices, in line with Eurotransplant guidelines [19], but some undocumented differences might exist. For DCD procurement, ventilator switch-off was done in the operating room or in the intensive care unit with rapid transportation of the donor to the operating theater after cessation of circulation. Heparin (300 U/kg) was administered before therapy withdrawal. Circulatory arrest was diagnosed according to local legislation and practices, followed by a period of five minutes (range 2 to 10 minutes) ‘no-touch period’ before declaration of death to rule out auto-resuscitation [20]. Cold preservation (i.e. cold ischemia time; CIT) was started by placing a canula in the abdominal aorta after rapid sterno-laparotomy [21] and flushing the vascular system with cold preservation solution. Three types of solution were used for both donor types according to local preferences, with a majority of DCD III organs preserved with histidine-tryptophan-ketoglutarate (HTK; 54% versus 23%, p < 0.001), a majority of DBD organs with University of Wisconsin solution (UW; 58% versus 29%, p < 0.001), and similar proportions with Institut George Lopez-1 solution (IGL-1; 19% and 17%). The agonal warm ischemia time (WIT) of DCD III was calculated from withdrawal of mechanical ventilation to circulatory arrest, and the acirculatory WIT was calculated from circulatory arrest to initiation of cold preservation (Fig 1). The mandatory no-touch is considered part of the acirculatory WIT. Pancreatectomy time was defined as the period between initiation of cold preservation and pancreatectomy.

**Table 1:** Donor and organ procurement characteristics.

	DCD III		DBD		*p*
n	141		609		
**Donor characteristics**					
Age (years)	52	(41–60)	54	(46–61)	0.097
Body mass index (kg/m^2^)	25	(23–28)	24	(23–27)	0.051
Gender (M/F)	64 / 36	46 / 54	<0.001
Cause of death (%)					<0.001
Anoxia / Cardiac arrest	36		9		<0.001
Trauma	28		26		1.000
Cerebrovascular / stroke	36		64		<0.001
Other			1		
Sodium (mmol/l)	145	(141–149)	148	(143–153)	<0.001
Glucose (mg/dl)	181	(139–223)	178	(144–232)	0.709
Lipase (U/l)	37	(20–65)	32	(19–73)	0.500
Alanine-aminotransferase (U/l)	57	(28–118)	33	(20–67)	<0.001
Lactate dehydrogenase (U/l)	412	(268–625)	391	(262–586)	0.311
Creatinin (μmol/l)	80	(63–109)	80	(62–102)	0.722
Time in hospital (days)	4	(2–7)	2	(1–5)	<0.001
Time in intensive care (days)	4	(2–6)	2	(1–4)	<0.001
Vasopressor use (%)	46		87		<0.001
Cardiac arrest (%)	45		24		<0.001
Hypotensive period (%)	21		36		<0.001
**Procurement characteristics**					
Procured by local team (%)	29		38		0.049
Preservation solution (%)					<0.001
IGL-1	17		19		1.000
UW	29		58		<0.001
HTK	54		23		<0.001
Pancreatectomy time (min)	46	(34–64)	43	(33–58)	0.072
Cold ischemia time (h)	6.9	(4.7–10.3)	7.4	(5.1–10.0)	0.369
**North American Islet Donor Score (%)**					<0.001
< 60	16		37		
60–70	33		30		
> 70	52		33		

Donor and organ procurement characteristics [represented as median (IQR) unless otherwise specified] of donors after controlled circulatory death (DCD III) and donors after brain death (DBD).

### Islet isolation, purification and culture

Islet cells were isolated using a modification of the automated Ricordi method [22]. During the study period, three types of enzyme were used for organ distention and digestion but only organs processed using NB1 collagenase (Serva Electrophoresis, Heidelberg, Germany) were included for analysis to exclude an effect of enzyme type on isolation outcome. Different batches of NB1 collagenase were evenly distributed between donor types, minimizing an effect of batch-to-batch variability on outcome. Isolates were purified by continuous gradient with Biocoll (Biochrom, Berlin, Germany) and a cooled COBE 2991 cell processor (Terumo BCT, Lakewood, CO, USA). After isolation and purification, the cell preparations were cultured in TC suspension culture flasks T175 (Sarstedt, Nümbrecht, Germany) at 37°C in a humidified incubator (5% CO_2_) in a Ham’s F10 based medium (Lonza, Bazel, Switzerland) or CMRL based medium (Mediatech, Manassas, VA, USA) [22].

### Quantification of islet isolation yield

Islet cell preparations were characterized by their number of beta cells after purification and after a 1- to 5-day culture period [determined after a minimum of two washing cycles to eliminate damaged cells and cell debris, by combining cell number (nuclear count assay in duplicate samples; NucleoCounter YC-100; ChemoMetec, Allerød, Denmark) and percentage of insulin positive cells (immunocytochemistry; >10^3^ cells counted)] [7, 22]. Yield immediately post purification was also expressed as islet equivalent (IEQ), calculated using a volume based method after dithizone (DTZ) staining [23, 24]. Only preparations with these data available after purification were included for analysis. Cellular insulin content was determined and expressed as μg insulin / 10^6^ beta cells [22].

### Statistical analysis

Individual characteristics on donor profile and pancreas procurement, as reported in the Eurotransplant donor and procurement file, as well as characteristics on pancreas processing, isolation yield and culture were stored in our Filemaker database (Filemaker Inc., Santa Clara, Ca, USA). If needed, missing data regarding ischemia time and type of cold preservation solution were requested at the donor center, bringing missing data to <5% for these key parameters. Results are presented as median [interquartile range (IQR)] if not otherwise specified. Statistical analysis was performed using SPSS (IBM, Armonk, NY, USA). Non-parametric tests [two tailed Mann-Whitney U test or Kruskal Wallis test for continuous variables, Chi-squared test for categorical variables, and Spearman’s rank correlation test for univariate analysis] were used for analysis. Pairwise comparisons after Kruskal Wallis or Chi-squared tests were performed after Bonferroni correction. Multivariate analysis was performed after rank transformation of variables. Statistical significance was assumed at *p < 0*.*05*.

## Results

### Comparison of islet isolation yield from DCD III organs with that after DBD

Pancreases from DCD III had a higher weight than organs procured from DBD, as well as larger post-digestion pellet volumes for a similar digestion time and percentage of undigested pancreas (Table 2). In DCD III, isolation yielded a lower beta cell number. This difference with DBD organs remained after a 1 to 5-day culture period. This was also the case when islet yield post-purification was assessed as IEQ after dithizone-staining. There were no differences in the respective percentages of insulin-positive cells and their cellular insulin content.

**Table 2:** Isolation procedure and islet cell isolate characteristics.

	DCD III		DBD		*p*
n	141		609		
**Isolation procedure**					
Pancreas weight (g)	107	(93–122)	98	(81–112)	<0.001
Digestion time (min >35°C)	14	(11–19)	14	(12–18)	0.935
Undigested pancreas (weight %)	20	(14–28)	22	(14–31)	0.139
Digest pellet volume (ml)	27	(20–32)	25	(20–30)	0.011
**Islet cell isolate**					
Beta cell purity (%)	24	(17–31)	24	(17–32)	0.615
Endocrine purity (% DTZ)	56	(48–65)	58	(48–70)	0.182
Beta cell number (x10^6^)					
after purification	58	(30–116)	84	(48–139)	<0.001
after 1–5 days culture	35	(15–60)	53	(30–84)	<0.001
IEQ (x 10^3^)					
after purification	98	(41–151)	130	(77–191)	<0.001
Insulin content (μg/10^6^ beta cells)					
after purification	17	(12–25)	19	(12–27)	0.274
after 1–5 days culture	13	(9–18)	14	(10–20)	0.047

Comparison of islet cell isolation outcome for pancreases procured from donors after controlled circulatory death (DCD III) and donors after brain death (DBD). Data are presented as median (IQR) unless otherwise specified, statistical significance (Mann-Whitney U) was assumed at *p < 0*.*05*.

### Correlation of donor and procurement variables with beta cell yield from DCD III organs in comparison with DBD

We examined whether beta cell yield was correlated with donor and procurement variables and whether this correlation was different between donor types (Table 3). For both DCD III and DBD islet cell isolates, multivariate analysis showed a positive correlation with donor body mass index and cold preservation solution. A negative correlation with plasma alanine-aminotransferase levels, and positive correlations with plasma lactate dehydrogenase levels and vasopressor use were seen in the DBD group, whereas the DCD III group exhibited a negative influence of male gender, plasma lactate dehydrogenase levels and acirculatory WIT. No correlation was observed with pancreatectomy time nor CIT. The negative correlation with acirculatory WIT was not associated with a statistically significant correlation to total WIT or agonal WIT. In univariate subgroup analysis, acirculatory WIT ≤ 10 minutes (cutoff selected using ROC–AUC), was associated with a higher beta cell yield than was the group with longer duration (Table 4). This was also the case after culture, when it was associated with a higher cellular insulin content.

**Table 3:** Correlations of donor and procurement variables with beta cell yield in islet cell isolates.

	DCD III				DBD			
	Univariate		Multivariate		Univariate		Multivariate	
	estimate	*(p)*	estimate	*(p)*	estimate	*(p)*	estimate	*(p)*
**Donor variables**								
Age	0.022	(0.799)	/	/	0.060	(0.137)	/	/
Body mass index	0.155	(0.067)	0.203	(0.012)	0.209	(<0.001)	0.222	(<0.001)
Male gender	-0.090	(0.288)	-0.166	(0.041)	-0.018	(0.661)	/	/
Cause of death	0.121	(0.153)	/	/	0.070	(0.086)	/	/
Sodium	-0.156	(0.065)	/	/	-0.064	(0.114)	/	/
Glucose	-0.092	(0.296)	/	/	-0.010	(0.813)	/	/
Lipase	-0.050	(0.644)	/	/	-0.065	(0.157)	/	/
Alanine-aminotransferase	-0.080	(0.348)	/	/	-0.060	(0.138)	-0.125	(0.003)
Lactate dehydrogenase	-0.168	(0.055)	-0.291	(0.001)	0.072	(0.085)	0.115	(0.007)
Creatinin	-0.171	(0.043)	/	/	0.031	(0.449)	/	/
Time in hospital	-0.073	(0.389)	/	/	-0.031	(0.449)	/	/
Time in intensive care	-0.115	(0.174)	/	/	-0.019	(0.632)	/	/
Vasopressor use	-0.094	(0.265)	/	/	0.083	(0.040)	0.096	(0.015)
Cardiac arrest	-0.026	(0.759)	/	/	-0.040	(0.327)	/	/
Hypotensive period	0.021	(0.801)	/	/	-0.054	(0.184)	/	/
**Procurement variables**								
Procured by local team	0.124	(0.142)	/	/	0.087	(0.032)	/	/
Preservation solution	0.200	(0.017)	0.219	(0.021)	0.092	(0.024)	0.117	(0.003)
Agonal WIT	-0.064	(0.464)	/	/	NA		NA	
Acirculatory WIT	-0.197	(0.021)	-0.234	(0.015)	NA		NA	
Total WIT	-0.137	(0.112)	/	/	NA		NA	
Pancreatectomy time	-0.125	(0.164)	/	/	-0.054	(0.188)	/	/
Cold ischemia time	-0.060	(0.477)	/	/	-0.008	(0.845)	/	/

Univariate (Spearman rank correlation test) and multivariate analysis (linear regression after rank transformation of variables) of correlation between donor and procurement variables and beta cell yield in islet cell isolates from pancreases after brain death (DBD) and after controlled circulatory death (DCD III). Data are presented as estimate (p-value). The estimate shows the correlation coefficient or standardized regression coefficient (ranges from -1 to 1 with a value approximating zero indicating no association). p-values are two sided and statistical significance was assumed at *p < 0*.*05*.

**Table 4:** Effect of acirculatory warm ischemia time in DCD III.

	Acirculatory WIT		Acirculatory WIT		*p*
	≤ 10 min		> 10 min		
n	68		70		
**Characteristics of islet cell isolate**					
Beta cell purity (%)	24	(17–31)	21	(15–30)	0.308
Endocrine purity (% DTZ)	57	(49–68)	53	(46–60)	0.184
Beta cell number (x10^6^)					
after purification	74	(37–128)	54	(13–94)	0.036
after 1–5 days culture	39	(22–67)	32	(10–52)	0.049
IEQ (x 10^3^)					
after purification	110	(55–156)	89	(31–138)	0.173
Insulin content (μg/10^6^ beta cells)					
after purification	17	(12–25)	19	(11–27)	0.485
after 1–5 days culture	15	(11–20)	11	(7–17)	0.015

Comparison of beta cell characteristics in islet cell isolates from DCD III-organs with acirculatory WIT duration up to 10 min and higher. Data are presented as median (IQR) unless otherwise specified, statistical significance (Mann-Whitney U) was assumed at *p < 0*.*05*. Missing data (n = 3).

### Use of IGL-1 preservation solution limits reduction in beta cell yield from DCD III organs

The observed correlation between beta cell yield and cold preservation solution was further examined by comparing outcome for the three used solutions in univariate subgroup analysis (Table 5). Use of IGL-1 was associated with a higher beta cell yield in islet cell isolates from both DCD III and DBD organs when compared with isolates from UW or HTK preserved pancreases. This difference remained after culture without an association with a higher beta cell purity and/or higher beta cell insulin content. The beneficial effect of IGL-1 in DCD III organs yielded beta cell numbers that were similar to those in DBD organs. To exclude a confounding effect of procurement by our local team that preferentially uses IGL-1, a subgroup analysis was performed. This confirmed our findings in both non-local procured DCD III organs [146 (65–289) x 10^6^ beta cells for IGL-1 versus 50 (29–63) x 10^6^ beta cells and 76 (14–129) x 10^6^ beta cells for UW and HTK; p = 0.030] and DBD organs [144 (72–263) x 10^6^ beta cells for IGL-1 versus 82 (47–125) x 10^6^ beta and 81 (36–122) x 10^6^ beta for UW and HTK; p = 0.013].

**Table 5:** Effect of cold preservation solution type on beta cell characteristics.

	DCD III						*p*	DBD						*p*
	UW		HTK		IGL-1			UW		HTK		IGL-1		
n	41		76		24			351		141		115		
**Characteristics of islet cell isolate**														
Beta cell purity (%)	21	(16–33)	24	(17–29)	28	(17–31)	0.631	24	(18–32)	23	(16–33)	23	(16–32)	0.789
Endocrine purity (% DTZ)	59	(50–70)	55	(47–62)	54	(47–67)	0.327	59	(47–70)	52	(47–67)	60	(50–68)	0.160
Beta cell number (x10^6^)														
after purification	54	(29–76)	65	(22–125)	86	(46–151)	0.042	82	(49–127)	81	(39–121)	108	(65–184)	<0.001
after 1–5 days culture	30	(14–42)	35	(11–63)	60	(25–91)	0.016	51	(30–81)	47	(25–74)	69	(41–115)	0.006
IEQ (x 10^3^)														
after purification	87	(42–128)	100	(34–155)	124	(61–173)	0.229	130	(79–185)	117	(72–174)	146	(86–228)	0.013
Insulin content (μg/10^6^ beta cells)														
after purification	19	(11–28)	17	(10–25)	18	(14–25)	0.634	19	(12–28)	17	(12–27)	18	(13–25)	0.603
after 1–5 days culture	11	(6–18)	13	(10–18)	14	(10–24)	0.210	15	(11–20)	14	(9–20)	14	(11–19)	0.743

Comparison of beta cell characteristics in islet cell isolates from DCD III and DBD-organs cold-preserved in UW, HTK or IGL-1. Data are presented as median (IQR) unless otherwise specified, statistical significance (Kruskal Wallis) was assumed at *p < 0*.*05*. Missing data (n = 2).

## Discussion

Scarcity of donor organs is a major limitation to clinical islet transplant programs. Less than half of the isolation procedures lead to islet cell preparations that meet criteria for clinical transplantation [6], and then often require additional injections of islet cell isolates [1]. In our clinical program islet cell isolates are cultured until quantitative criteria are met for an injection. This pretreatment removes acinar cells as well as damaged cells and debris. It is also associated with a loss of beta cells but not with a reduction in their percentage [22], and it can be used to combine preparations if quantitative criteria are not immediately met, while preserving the desired metabolic effect in patients [8]. However, since beta cell recovery after culture may be influenced by many confounders [22, 25, 26], the measurement of beta cells after purification was used to examine if DCD III organs are suitable for the preparation of islet cell grafts. Islet mass serves since long as a criterium to select pancreatic islet isolates for clinical transplantation [1, 6, 15]. It is usually determined as the number of IEQ after dithizone staining [27]. Since the intensity of this staining decreases during culture in absence of a loss of viable beta cells, we have replaced it by a count of beta cells, which, in addition, is a direct assessment of the active component in the grafts. We, and others, do not perform functional beta cell assays on each isolate [15]. This is not to deny the importance of the functional state of the prepared beta cell mass for its therapeutic use; it reflects the lack of time to add the data of this analysis to the selection process [6].

Proof of principle to use organs from donors after circulatory death as additional source for islet cell transplantation was already shown previously. While some reports included donors where circulatory arrest occurs under uncontrolled circumstances (DCD category II) and that are subject to long and not well documented periods of warm ischemia [28–30], the remaining cohorts involved highly selected DCD III [10, 11, 16, 17].

The organs that we receive from the Eurotransplant network are heterogenous in quality and in procurement conditions. This led us to analyze correlations between beta cell yield and donor and procurement variables. At variance with previous reports [10, 11, 16, 17], we observed a lower isolation yield from DCD III than from DBD pancreases, with in average thirty percent less beta cells after isolation and after culture. This finding is however in line with observations in other fields of organ transplantation where DCD III organs are considered to be at higher risk for posttransplant dysfunction and complications due to longer warm ischemia [12–14]. During review of this manuscript, the Leiden group demonstrated a similar reduction in islet cell yield from DCD III organs [31].

The effects of warm ischemia on islet isolation have been previously studied in animal models where quantitative (islet counts) and qualitative outcome parameters (glucose stimulated insulin release) decreased with increasing WIT [32]. In our cohort of DCD III-organs total WIT ranged from 8 minutes to over 1.5 hours, but we did not measure a negative correlation with the number of beta cells in the isolates. For human donor organs, the agonal phase of total WIT is known to exhibit a high variability in its consequences on organ perfusion and oxygenation [33]. We therefore examined the effects of acirculatory WIT, the time between circulatory arrest and start of cold preservation, which varied between 3 and 36 minutes. The duration of acirculatory WIT was found to independently affect isolation outcome determined by the number of beta cells in our study. When acirculatory WIT did not exceed 10 minutes, beta cell yield was not impaired, an effect that was maintained during culture and associated with a better preservation of cellular insulin content. These data suggest that acirculatory WIT up to 10 minutes can be used to select DCD III-organs for islet cell isolation. Our study does however not provide direct proof for the higher clinical efficacy of their beta cell preparations as compared to those with longer acirculatory WIT. A negative clinical outcome of donor tissue with longer acirculatory WIT has been reported for kidney transplants [34]. Contribution of other ischemic insults has also more impact on posttransplant outcome in DCD III organs as was previously shown for kidney transplants [35]. This is in line with our observation of a negative correlation between serum lactate dehydrogenase levels, a marker for ischemic organ damage before organ recovery [36] and beta cell yield in DCD III but not in DBD-organs.

In line with previous observations within the United Network for Organ Sharing, HTK was used for a majority of our DCD III organs [37]. However, use of DCD III pancreases for islet cell isolation benefits from their cold preservation in IGL-1. This solution appeared independently associated with a higher beta cell yield, bringing the numbers to those obtained for DBD-organs. It has been previously reported that IGL-1 was not inferior to UW when evaluating islet isolation from DBD organs with short CIT [38]. Several studies demonstrated benefits of IGL-1 in preserving abdominal organs including those with long preservation time or ischemic injury [39–41]. When compared to UW, the protective effect of IGL-1 has been attributed to its low potassium-sodium ratio and its markedly lower viscosity, due to the use of polyethylene glycol as a colloid instead of hydroxyethyl starch, which is expected to enable higher flow rates and thus a better microcirculation [42]. The use of polyethylene glycol has also been shown to reduce lipid peroxidation [43, 44] and preserve mitochondrial integrity [45], both effects that could be beneficial in the setting of islet cell transplantation.

In conclusion, DCD III pancreases, procured within the Eurotransplant network, yielded a lower number of beta cells compared to DBD. This lower beta cell yield was prevented by limiting initial acirculatory warm ischemia time and using IGL-1 as cold preservation solution.
